# Effects of *DSM-5* Betel-Quid-Related Symptoms, Pathological Behaviors, and Use Disorder on Oral Squamous Cell Carcinoma Risk

**DOI:** 10.3390/cancers14163974

**Published:** 2022-08-17

**Authors:** Wen-Chen Wang, Yueh-Tzu Chiu, Yen-Yun Wang, Shuai-Lun Lu, Leong-Perng Chan, Chun-Ying Lee, Frances M. Yang, Shyng-Shiou F. Yuan, Chien-Hung Lee

**Affiliations:** 1 School of Dentistry, College of Dental Medicine, Kaohsiung Medical University, Kaohsiung 80708, Taiwan; 2 Division of Oral Pathology and Oromaxillofacial Radiology, Department of Dentistry, Kaohsiung Medical University Hospital, Kaohsiung 80756, Taiwan; 3 Department of Public Health, College of Health Sciences, Kaohsiung Medical University, Kaohsiung 80708, Taiwan; 4 Department of Medical Research, Kaohsiung Medical University Hospital, Kaohsiung 80756, Taiwan; 5 Faculty of Medicine, College of Medicine, Kaohsiung Medical University, Kaohsiung 80708, Taiwan; 6 Department of Otorhinolaryngology-Head and Neck Surgery, Kaohsiung Municipal Ta-Tung Hospital, Kaohsiung 80145, Taiwan; 7 Cohort Research Center, Kaohsiung Medical University, Kaohsiung 80708, Taiwan; 8 Department of Family Medicine, Kaohsiung Medical University Hospital, Kaohsiung 80756, Taiwan; 9 School of Nursing, KU Medical Center, The University of Kansas, Kansas City, KS 66103, USA; 10 Graduate Institute of Medicine, College of Medicine, Kaohsiung Medical University, Kaohsiung 80708, Taiwan; 11 Department of Obstetrics and Gynecology, Kaohsiung Medical University Hospital, Kaohsiung 80756, Taiwan; 12 Department of Biological Science and Technology, Institute of Molecular Medicine and Bioengineering, Center for Intelligent Drug Systems and Smart Bio-Devices (IDS2B), National Yang Ming Chiao Tung University, Hsinchu 30009, Taiwan; 13 Research Center for Environmental Medicine, Kaohsiung Medical University, Kaohsiung 80708, Taiwan; 14 Office of Institutional Research & Planning, Secretariat, Kaohsiung Medical University, Kaohsiung 80708, Taiwan

**Keywords:** betel quid, substance use disorder, oral cancer, *DSM-5* symptoms, pathological behavior

## Abstract

**Simple Summary:**

This is the first study using psychometrics to investigate the association of the *DSM*-*5* betel-quid (BQ)-related symptoms, pathological behaviors, and BQ use disorder (BUD) severity with the risk of oral squamous cell carcinoma (OSCC). This study presents data demonstrating that the symptoms of unsuccessful cutdown of BQ consumption, neglecting major roles, social or interpersonal problems, abandoning or limiting activities, hazardous use, and continued use despite awareness of the dangers were associated with the risk of OSCC. Pathological behavior of risky BQ use enhanced OSCC risk in chewers with moderate-to-severe BUD. BQ chewing is a major risk factor for oral cavity cancers; however, the risk can be mitigated by reducing pathological use. Our study indicates that targeting BUD and establishing a BUD-based strategy is a promising new direction for the prevention of OSCC.

**Abstract:**

The neuroactive alkaloids in betel quid (BQ) can induce BQ addiction. We conducted a case–control study to investigate the effects of BQ-associated *DSM-5* symptoms, pathological behaviors, and BQ use disorder (BUD) on oral squamous cell carcinoma (OSCC) risk. A total of 233 patients with newly diagnosed and histopathologically confirmed OSCC and 301 sex- and age-matched controls were included. BQ-related *DSM-5* symptoms in the 12 months prior to disease onset were used to measure psychiatric characteristics and BUD. Compared with nonchewers, chewers with the symptoms of unsuccessful cutdown of BQ consumption, neglecting major roles, social or interpersonal problems, abandoning or limiting activities, hazardous use, and continued use despite the awareness of the dangers had a 54.8-, 49.3-, 49.9-, 40.4-, 86.2-, and 42.9-fold higher risk of developing OSCC, respectively. Mild-to-moderate and severe BUD were, respectively, associated with a 8.2–8.5- and 42.3-fold higher OSCC risk, compared with BQ nonuse. Risky BQ use of pathological behavior was associated with a 12.5-fold higher OSCC risk in chewers with no BUD or mild BUD and a 65.0-fold higher risk in chewers with moderate-to-severe BUD (*p* for risk heterogeneity between the two BUD groups, 0.041). In conclusion, BQ-associated *DSM-5* symptoms, pathological behaviors, and BUD severity are associated with the impact of BQ chewing on OSCC development. The pathological behavior of risky BQ use enhances OSCC risk in chewers with moderate-to-severe BUD. Preventing BUD in new BQ users and treating BUD in chewers who already have the disorder are two priorities in areas where BQ chewing is prevalent.

## 1. Introduction

Betel quid (BQ) is an areca nut (AN)-based mixture, usually prepared by wrapping betel leaves coated with slaked lime around an AN and selected ingredients, such as tobacco, spices, and the flower of the *Piper betle* [1,2]. In regions where BQ chewing is prevalent, BQ can be purchased inexpensively in ready-made packaging [3]. Over 600 million people are estimated to chew BQ worldwide, primarily in South and Southeast Asia and among Asian immigrant populations in the United Kingdom and United States [4].

The expert working groups arranged by the International Agency for Research on Cancer have classified the AN itself and BQ (with or without tobacco) as Group 1 human carcinogens [5,6,7]. Arecoline is the major active alkaloid in the AN [8]. The carcinogenesis of oral squamous cell carcinoma (OSCC) is linked to arecoline-induced genotoxic effects, reactive oxygen species derived from the interaction of slaked lime with the AN, and the formation of nitrosamines [2,9,10,11]. Epidemiological studies have specified that BQ chewing is a significant risk factor for the development of aerodigestive tract cancers at four anatomical sites (the oral cavity, pharynx, esophagus, and larynx) [12,13,14,15]. The health hazards presented by BQ chewing are a major public concern in areas where this substance is habitually used.

The AN contains four neuroactive alkaloids, namely, arecoline, guvacoline, guvacine, and arecaidine, all of which have pharmacological action and psychological effects [1,16]. Arecoline and guvacoline are muscarinic agonists, and arecoline has a chemical structure similar to that of nicotine [17,18]. Guvacine and arecaidine are gamma-aminobutyric acid uptake inhibitors, which increase the synaptic availability of gamma-aminobutyric acid [19,20]. The pharmacological and psychological profiles of these alkaloids can cause BQ addiction among chewers. In resting-state functional magnetic resonance imaging studies, BQ chewing has been observed to acutely influence the functional connectivity of the frontal and default networks, which are known to be critical in addiction [21,22].

In a study conducted by the Asian Betel-Quid Consortium in Taiwan, Mainland China, Malaysia, Indonesia, Nepal, and Sri Lanka, the prevalence of BQ abuse and dependence was 0.8–46.3% and 2.8–39.2%, respectively, and the addictive use of BQ was associated with oral potentially malignant disorders (OPMDs) [23,24,25]. The same investigation indicated that the BQ-associated symptoms derived from the *Diagnostic and Statistical Manual of Mental Disorders*, *Fifth Edition* (*DSM-5*) exhibit sufficient unidimensionality as valid indicators of BQ use disorder (BUD). Among individuals with moderate-to-severe BUD, a larger amount of BQ usage and BQ tolerance are major symptoms linked to a strengthened risk for OPMD [26]. The *DSM-5* BUD construct includes eleven symptoms and four pathological behaviors and divides BUD into four levels of severity [27]. How addictive BQ use behaviors affect OSCC risk remains undetermined. Accordingly, we conducted a case–control study to investigate the effects of BQ-associated *DSM-5* symptoms, pathological behaviors, and BUD on the risk of OSCC.

## 2. Patients and Methods

### 2.1. Patient Data

This hospital-based case–control study was conducted in Kaohsiung, Taiwan. All research protocols and procedures were reviewed and approved by the Institutional Review Board of Kaohsiung Medical University Hospital (KMUH). Patients with and without OSCC were recruited from KMUH, which is a highly regarded medical center in southern Taiwan serving patients from all socioeconomic groups. This study enrolled patients with suspected primary invasive carcinoma of the oral cavity who visited the Department of Oral and Maxillofacial Surgery, Special Outpatient Clinic for Oral Pathology, or Department of Otolaryngology at KMUH between April 2016 and April 2018. Cells and histological specimens were obtained from the participants and then sent to the Department of Pathology for pathological confirmation of OSCC. The inclusion criteria for the OSCC group were (1) age of 18 years or older and (2) Taiwanese nationality. The exclusion criteria were (1) a concurrent diagnosis of another type of tumor and (2) past history of cancer. After 16 patients who either refused to participate or submitted incomplete data were excluded, 233 patients with OSCC were enrolled in this study.

The control individuals were recruited from the Health Management Center, Department of Family Medicine, and Department of Gastroenterology at KMUH during the same period as the OSCC group recruitment. The Health Management Center provides regular medical checkups for the general public. In a case–control study, the inclusion and exclusion criteria for the control group are generally the same as those used for the case group. However, in the present study, patients with any type of OPMD or oral tumor and those with disorders highly correlated with smoking were excluded. Within 4 weeks of enrolling each patient with OSCC, we began to search for sex- and age- (within 3 years) matched controls. The first and second eligible controls to be identified were invited to participate in this study. After 11 patients with chronic obstructive pulmonary disease, 2 patients with oral cancer, and 1 patient with incomplete data were excluded, 301 control patients were included in the study. This investigation was conducted in accordance with the principles of the Declaration of Helsinki. We obtained written informed consent from each participant in both the OSCC and control groups.

### 2.2. Data Collection

A structured questionnaire predesigned specifically for this investigation was used to collect data on the participants’ sociodemographics, occupations, previous diseases, medication histories, substance use histories, and BQ-associated *DSM-5* symptoms. All interviews for both study groups were conducted by trained interviewers. BQ chewers were defined as those who had chewed a minimum of one BQ daily for at least 6 months. The type of BQ used was recorded as AN only, AN wrapping betel leaf, AN adding flower of the *Piper betle*, and mixed use. Alcohol drinkers were defined as those who had consumed a minimum of one alcoholic beverage per week for at least 6 months; one drink, which is equivalent to drinking 350 mL of beer with 5.0% alcohol by volume, contains 17.5 g of pure ethanol. Cigarette smokers were considered to be those who had smoked a minimum of one cigarette per day for at least 6 months. To examine the effects of cumulative lifetime exposure to BQ, alcohol, and cigarettes on the risk of OSCC, we, respectively, calculated BQ pack × years, alcohol drink × years, and cigarette pack × years by multiplying the number of BQ packs (10 BQ) chewed, alcoholic drinks (17.5 g alcohol) consumed, and cigarette packs (20 cigarettes) smoked per day by the number of years that the participants had used the substance.

### 2.3. Measurement of BQ-Related DSM-5 Symptoms

The *DSM-5* measurement scale, which was applied by the Asian Betel-Quid Consortium for its OPMD study, was used to evaluate BUD [26]. The scale measures 11 BQ-related *DSM-5* symptoms as reported by BQ chewers during the 12 months prior to disease onset. The symptoms of *DSM-5* BUD and their meanings were displayed in Table 1. These symptoms are divided into four categories of pathological behavior: (1) impaired control (symptoms 1–4), (2) social impairment (symptoms 5–7), (3) risky use (symptoms 8 and 9), and (4) pharmacologic indicator (symptoms 10 and 11). According to the *DSM-5* criteria for substance use disorders, zero or one symptom indicates no BUD, two or three symptoms indicate mild BUD, four or five symptoms indicate moderate BUD, and six or more symptoms indicate severe BUD [27,28].

### 2.4. Statistical Analysis

Proportions and means with standard deviations were employed to describe the distributions of categorical and continuous variables, respectively. The associations between sociodemographic characteristics and cancer status were examined using a chi-square test. Because the effect of BQ use on OSCC risk was the primary focus of this study, potential confounders were evaluated through the changes in odds ratio (OR) of OSCC associated with BQ use between the original model and the model with the added confounder. Factors with a >10% change in OR and those identified in the literature as risk factors for OSCC were considered confounders [29,30]. In this study, sex, age, education level, occupation, income, alcohol drinking, and cigarette smoking were considered confounders. All risk estimates were adjusted for the effects of these variables in multivariable regression models. We used logistic regression models to evaluate the associations of cumulative lifetime exposure to BQ, alcohol, and cigarettes with OSCC development. The linear trends in the adjusted odds ratio (aOR) of ordinal exposure levels were investigated by scoring the exposure variable and treating the scoring variable as continuous. We applied multivariable logistic regression models to assess the effects of BQ-related *DSM-5* symptoms, pathological behaviors, and BUD on OSCC risk. The potential combined, conditional, and heterogeneous interaction effects of pathological behavior and BUD on OSCC risk were evaluated [26]. The combined effect referred to a synergistic effect of pathological behavior and BUD status on OSCC; the conditional effect was an effect of pathological behavior on OSCC, conditional on BUD status; and the heterogeneous effect was assessed using an aOR ratio indicating the difference in the conditional aORs between two groups of different BUD status.

## 3. Results

### 3.1. Demographic Distribution and Cancer Status

Table 2 displays the distribution of sociodemographic factors for the patients with OSCC and the controls. Larger proportions of the patients with OSCC than of the controls had ≤9 years of education (49.4% vs. 13.6%) and monthly incomes of <20,000 new Taiwan dollar (51.5% vs. 25.3%). Higher proportions of the patients with OSCC than of their control counterparts were unemployed or retired (50.6% vs. 29.6%) or skilled workers (18.5% vs. 10.6%).

### 3.2. Cumulative Lifetime Exposure of Subtance and OSCC Risk

Table 3 reveals the adjusted risk of OSCC associated with cumulative lifetime consumption of BQ, alcohol, and cigarettes. Greater lifetime BQ chewing, alcohol drinking, and cigarette smoking were associated with a greater risk of developing OSCC (all linear trends of cancer risk were significant, *p* < 0.001, Model 1). After adjustment for the effects of covariates and use of other substances, participants with >20 pack × years of BQ chewing, >20 drink × years of alcohol drinking, and >20 pack × years of cigarette smoking had a 19.4-, 3.1-, and 3.2-fold higher OSCC risk than those of nonusers (Model 2).

### 3.3. Effect of DSM-5 Symptoms, Pathological Behavior, and BUD on OSCC Risk

Table 4 presents the effects of *DSM-5* symptoms, pathological behavior, and BUD on OSCC. Compared with BQ nonchewers, chewers with the symptoms of unsuccessful cutdown of BQ consumption, neglecting major roles, social or interpersonal problems, abandoning or limiting activities, hazardous use, and continued use despite the awareness of the dangers had a >40-fold higher OSCC risk (aORs of 54.8, 49.3, 49.9, 40.4, 86.2, and 42.9, respectively). Among pathological behaviors, each additional impaired control, social impairment, risky use, and pharmacologic indicator were correspondingly associated with a 2.3-, 8.2-, 9.6-, and 3.4-fold greater OSCC risk. BQ chewers without BUD had a 5.7-fold OSCC risk; however, chewers with mild, moderate, and severe BUD had an 8.5-, 8.2- and 42.3-fold greater risk of OSCC, respectively, compared with nonchewers. The majority of BQ chewers used the AN-wrapped betel leaf type (76.2% for OSCC and 55.2% for the control group), and 15.8% of the chewers were mixed-type users (Supplementary Table S1). BUD chewers had a 21.1-fold higher risk of OSCC among chewers using the AN-wrapped betel leaf type, and 12.7-fold higher risk of OSCC among chewers using other types of BQ (Supplementary Table S2).

### 3.4. BQ-Related DSM-5 Symptoms and OSCC Risk in BQ Chewers

The influence of BQ-related *DSM-5* symptoms and pathological behaviors on OSCC risk in BQ chewers is displayed in Table 5. The symptoms of unsuccessful cutdown of BQ consumption, neglecting major roles, social or interpersonal problems, abandoning or limiting activities, hazardous use, continued use despite the awareness of the dangers, and withdrawal were associated with a >3.0-fold greater risk of OSCC (aORs of 9.7, 3.2, 8.8, 5.0, 13.8, 6.6, and 3.1, respectively). Social impairment and risky use were significantly associated with a higher OSCC risk among chewers. Chewers with severe BUD had a 7.7-fold higher OSCC risk than those without BUD.

### 3.5. Combined Effects of DSM-5-Defined BUD and Pathological Behaviors on OSCC

Table 6 reveals the combined effects of *DSM-5*-defined BUD and pathological behaviors on OSCC. Compared with nonchewers, BQ chewers with moderate-to-severe BUD and at least one impaired control, social impairment, risky use, or pharmacologic indicator had a 23.7-, 32.0-, 65.0-, and 19.3-fold risk of developing OSCC, respectively. Among BQ chewers with no BUD or mild BUD, those with risky use had a 2.2-fold greater conditional OSCC risk compared with those without; among chewers with moderate-to-severe BUD, this conditional OSCC risk was 23.9-fold. Furthermore, the aOR ratio of the conditional aOR of risky BQ use by chewers with moderate-to-severe to that of risky BQ use by chewers with no BUD or mild BUD was 10.9, representing heterogeneous conditional (stratified) aORs between the two BUD groups (*p* for risk heterogeneity = 0.041).

## 4. Discussion

This study presents data demonstrating that OSCC risk is closely related to the symptoms and pathological behaviors of BQ chewers indicated in the *DSM-5*. Specifically, BQ users with severe BUD have an increased risk of developing OSCC.

A comprehensive meta-analysis of 45 case–control studies and 8 cohort studies estimated that the relative risk of oral cancer for tobacco-free BQ chewing was 7.9, with the population attributable risk burden for this substance being 53.7% for Taiwan and 49.5% for Indian populations [2]. In our study, BQ chewers had a 12.6-fold higher risk of OSCC, and chewers with >20 pack × years of cumulative lifetime use had a 19.4-fold greater risk. These data emphasize the causal role of BQ use in the development of oral cancer. In a systematic review and meta-analysis of data from 2003–2017, Yang et al. discovered that BQ use was associated with worse prognosis among patients with oral cancer; BQ users had a pooled hazard ratio of 1.3 for 5-year all-cause mortality versus nonusers [31].

This study identified significant risks of OSCC among alcohol and tobacco users, discovering a dose–response relationship between cumulative lifetime use and cancer risk. These two substances are the main risk factors for oral cavity cancers in countries where BQ is rarely used [15,32,33]. In countries where BQ chewing is common, BQ is frequently used along with alcohol and tobacco/cigarettes [3]. Several epidemiological investigations have observed the combined use of alcohol, BQ, and cigarettes to be strongly associated with an increased risk of OSCC (estimated risks at 39.7–122.8-fold) [34,35,36]. These findings indicate the importance of combined alcohol, BQ, and tobacco use prevention and intervention programs for reducing OSCC occurrence.

Epidemiological studies have observed that chronic BQ chewers demonstrate specific dependency symptoms, including several associated with BQ additives [24,25,37,38]. In an international survey of BQ chewers in six Asian regions, BQ chewers in Nepal and Indonesia had noticeable tolerance and withdrawal symptoms, BQ chewers in Sri Lanka and Malaysia demonstrated cravings, and BQ chewers in Taiwan and Hunan, spent considerable time chewing BQ [24,25]. An Indian study discovered that BQ chewers, both those who smoke and do not smoke, exhibited clear withdrawal symptoms and craving-like effects [37]. Using the *DSM-5* criteria to measure BUD and BQ addition, this study further revealed that mild-to-moderate BUD was associated with an 8.2–8.5-fold higher risk of OSCC, compared with nonchewing, and severe BUD was associated with a 42.3-fold higher risk. Our data underscore the importance of treating BUD, particularly severe BUD, for reducing oral cancer; therefore, prevention strategies should focus on preventing new BQ chewers from developing BUD, and intervention strategies should focus on clinically treating BQ chewers with BUD.

Our study revealed that BQ chewers with the pathological behaviors of risky use and social impairment had a notably higher risk of OSCC, with risky use having a greater effect than social impairment (aOR for each additional symptom, 9.6 vs. 8.2). This finding implies that the psychiatric characteristics of continuing BQ chewing despite physical disorders or awareness of the physical or psychological hazards were useful for identifying chewers with high OSCC risks. In this investigation, BQ chewers with risky use had higher average amounts of daily use and years of use than nonrisky users had (amount of use: 37.1 vs. 22.0 BQ/day; years of use: 28.2 vs. 23.3 years, both *p* ≤ 0.0014). Alternatively, studies on head and neck squamous cell carcinomas have reported a relationship between cancer development and distress; specifically, 56–58% of such patients exhibit clinically significant distress [39,40]. Our study revealed that BQ-associated social impairment is associated with a higher OSCC risk. Social impairment might affect cancer development by increasing social distress.

In a representative investigation of six populations with endemic BQ chewing conducted by the Asian Betel-Quid Consortium, BQ tolerance and a larger amount/longer history of BQ chewing were symptoms linking to an increased OPMD risk for BQ chewers with moderate-to-severe BUD [26]. In this study, risky use was the pathological behavior most strongly correlated with OSCC risk in moderate-to-severe BUD chewers (conditional aORs for risky use among chewers with moderate-to-severe BUD and those with no BUD or mild BUD of 23.9 and 2.2, respectively, with an aOR ratio of 10.9). These data suggest that the *DSM-5* psychiatric characteristics associated with oral precancer and cancer are not identical. Risky use is a notable psychiatric feature that involves continued BQ chewing despite BQ-induced diseases or an understanding of the risks of such diseases. This psychiatric feature can help clinicians identify BQ chewers with BUD who have a high risk of OSCC.

This study has several strengths. This is the first study using psychometrics to investigate the association of the *DSM-5* BQ-related symptoms, pathological behaviors, and BUD severity with the risk of OSCC. Our research methodology can be applied in countries with endemic BQ chewing to examine the effect of BQ-associated psychiatric characteristics on the development of OSCC. We considered the effects of several major confounding factors in our risk evaluations. However, we note two limitations. First, recall bias in the BQ-related *DSM-5* symptoms data might be present. Our data were based on patients’ recent memories (12 months before the onset of OSCC or corresponding control illness), and with a range of diseases employed as the control group (multiple controls), the impact of this potential bias on the results should be limited. Second, our study was not a large-scale investigation; therefore, further research is needed to corroborate our findings. Nevertheless, our study indicates that targeting BUD and establishing a BUD-based strategy is a promising new direction for the prevention of OSCC.

## 5. Conclusions

BQ-associated *DSM-5* symptoms, pathological behaviors, and BUD severity were closely associated with the impact of BQ chewing on OSCC development. Pathological risky BQ use is associated with increased OSCC risk in chewers with moderate-to-severe BUD. This study has two translational values. First, BQ chewing is a major risk factor for oral cavity cancers; however, the risk can be mitigated by reducing pathological use in BUD chewers, particularly severe BUD. Second, preventing BUD in new BQ users and clinically treating BUD in chewers who already have the disorder are two priorities in areas where BQ chewing is prevalent.

## Figures and Tables

**Table 1:** The betel-quid-related *DSM-5* symptoms and meanings.

Symptoms	Meanings
Larger amount of intake	A larger amount of betel quid chewing than intended
2. Unsuccessful cutdown	Unsuccessful cutdown of betel quid chewing
3. Time spent using betel quid	Spending considerable time chewing betel quid
4. Craving	Having strong cravings to chew betel quid
5. Neglecting major roles	Failing to fulfill major role obligations at work or home as a result of recurrent betel quid chewing
6. Social or interpersonal problems	Continually chewing betel quid despite persistent or recurrent social or interpersonal problems caused or exacerbated by the effects of betel quid chewing
7. Abandoning or limiting activities	Abandoning or limiting important social, occupational, or recreational activities because of betel quid chewing
8. Hazardous use	Repeatedly chewing betel quid in situations in which it is physically hazardous
9. Continued use despite knowing problems	Continued betel quid chewing despite the awareness of the physical or psychological problems caused by chewing
10. Tolerance	Having betel quid tolerance symptoms
11. Withdrawal	Having betel quid withdrawal symptoms

**Table 2:** The distribution of demographic factors in oral squamous cell carcinoma patients and controls.

	OSCC (N = 233)		Control (N = 301)		
Factors	No.	%	No.	%	*p* ^a^
**Sex**					
Female	5	2.2	8	2.7	0.703
Male	228	97.9	293	97.3	
**Age, year**					
≤34	2	0.9	5	1.7	0.389
35–44	42	18.0	70	23.3	
45–54	73	31.3	85	28.2	
≥55	116	49.8	141	46.8	
**Ethnicity**					
Minnan	222	95.3	280	93.0	0.215
Mainlander	3	1.3	10	3.3	
Hakka	4	1.7	9	3.0	
Aboriginal	4	1.7	2	0.7	
**Marital status**					
Unmarried	30	12.9	39	13.0	0.978
Married	203	87.1	262	87.0	
**Educational level, year**					
≤9	115	49.4	41	13.6	<0.001
10–12	96	41.2	88	29.2	
>12	22	9.4	172	57.1	
**Income, NTD**					
<20,000	120	51.5	76	25.3	<0.001
20,000–39,999	61	26.2	72	23.9	
40,000–59,999	32	13.7	78	25.9	
≥60,000	20	8.6	75	24.9	
**Occupation**					
Administrative staff	22	9.4	110	36.5	<0.001
Labor workers	27	11.6	31	10.3	
Skilled workers	43	18.5	32	10.6	
Salesperson	23	9.9	39	13.0	
Unemployed or retired	118	50.6	89	29.6	

OSCC, oral squamous cell carcinoma; NTD, new Taiwan dollar. ^a^ *p* values for the associations between demographic factors and OSCC were obtained from the chi-squared test.

**Table 3:** Adjusted odds ratios of oral squamous cell carcinoma associated with substance use.

	OSCC (N = 233)		Control (N = 301)		Model 1 ^a^		Model 2 ^b^	
Substances	No.	%	No.	%	aOR	(95% CI)	aOR	(95% CI)
**Betel quid chewing, pack × years**								
Nonchewer	40	17.2	272	90.4	1.0		1.0	
Chewer	193	82.8	29	9.6	25.4	(14.3–45.2)	12.6	(6.8–23.4)
≤20	69	29.6	19	6.3	15.7	(7.9–31.1)	8.7	(4.2–17.9)
>20	124	53.2	10	3.3	43.3	(19.7–95.3)	19.4	(8.4–45.2)
*p* for linear trend					<0.001		<0.001	
**Alcohol drinking, drink × years**								
Non-drinker	74	31.8	257	85.4	1.0		1.0	
Drinker	159	68.2	44	14.6	7.8	(4.7–12.8)	3.1	(1.7–5.7)
≤20	26	11.2	13	4.3	5.0	(2.1–11.9)	3.1	(1.1–9.2)
>20	133	57.1	31	10.3	8.9	(5.1–15.4)	3.1	(1.6–6.1)
*p* for linear trend					<0.001		<0.001	
**Cigarette smoking, pack × years**								
Non-smoker	33	14.2	208	69.1	1.0		1.0	
Smoker	200	85.8	93	30.9	8.0	(4.8–13.4)	2.8	(1.5–5.3)
≤20	43	18.5	42	14.0	4.8	(2.4–9.3)	1.9	(0.8–4.3)
>20	157	67.4	51	16.9	10.3	(5.9–18.0)	3.2	(1.6–6.5)
*p* for linear trend					<0.001		0.002	

OSCC, oral squamous cell carcinoma; aOR, adjusted odds ratio. ^a^ In model 1, aORs were adjusted for sex, age, educational level, income, and occupation. ^b^ In model 2, aORs were adjusted for the covariates in the model 1, as well as pack (or drink) years of betel quid chewing, alcohol drinking, and cigarette smoking, where appropriate.

**Table 4:** Adjusted odds ratios of oral squamous cell carcinoma associated with *DSM-5* symptoms, pathological behavior, and use disorder of betel quid.

	OSCC (N = 233)		Control (N = 301)			
*DSM*-*5* Parameters	No.	(%)	No.	(%)	aOR ^a^	(95% CI)
Betel quid chewing						
Nonchewer	40	(17.2)	272	(90.4)	1.0	Ref.
Chewer	193	(82.8)	29	(9.6)		
**Symptom in chewers**						
1. Larger amount of intake	109	(46.8)	11	(3.7)	14.9	(6.5–34.2)
2. Unsuccessful cutdown	97	(41.6)	4	(1.3)	54.8	(17.4–172.6)
3. Time spent using betel quid	129	(55.4)	13	(4.3)	17.8	(8.1–39.1)
4. Craving	124	(53.2)	10	(3.3)	19.4	(8.2–45.7)
5. Neglecting major roles	31	(13.3)	1	(0.3)	49.3	(5.6–437.5)
6. Social or interpersonal problems	121	(51.9)	5	(1.7)	49.9	(17.3–144.2)
7. Abandoning or limiting activities	87	(37.3)	4	(1.3)	40.4	(12.7–128.4)
8. Hazardous use	92	(39.5)	2	(0.7)	86.2	(18.5–401.1)
9. Continued use despite knowing problems	127	(54.5)	6	(2.0)	42.9	(15.7–117.0)
10. Tolerance	111	(47.6)	10	(3.3)	18.8	(8.1–43.6)
11. Withdrawal	117	(50.2)	9	(3.0)	21.2	(8.8–51.2)
**Symptom no. of pathological behavior, mean ± SD**						
Impaired control, no.	2.4 ± 1.5		1.3 ± 1.3		2.3	(1.8–3.0)
Social impairment, no.	1.2 ± 1.0		0.3 ± 0.6		8.2	(4.1–16.5)
Risky use, no.	1.1 ± 0.8		0.3 ± 0.6		9.6	(4.5–20.7)
Pharmacological indicator, no.	1.2 ± 0.9		0.7 ± 0.8		3.4	(2.2–5.3)
**Betel quid use disorder**						
None (0–1 symptoms)	40		272		5.7	(2.4–13.4)
Positive	32		13			
Mild (2–3 symptoms)	22		6		8.5	(2.8–25.5)
Moderate (4–5 symptoms)	27		5		8.2	(2.6–26.2)
Severe (≥6 symptoms)	112		5		42.3	(14.3–125.0)

*DSM*-*5*, Diagnostic and Statistical Manual of Mental Disorders, 5th Edition; OSCC, oral squamous cell carcinoma; aOR, adjusted odds ratio; Ref., reference group. ^a^ aORs were adjusted for sex, age, educational level, income, and occupation, drink × years of drinking and pack × years of smoking.

**Table 5:** Adjusted odds ratios of oral squamous cell carcinoma associated with *DSM*-*5* symptoms among betel quid chewers.

	OSCC (N = 193)				Control (N = 29)						
*DSM*-*5* Parameters	Yes		No		Yes		No				
	No.	(%)	No.	(%)	No.	(%)	No.	(%)	aOR ^a^	(95% CI)	*p* ^a^
**Chewers with symptom**											
1. Larger amount of intake	109	(56.5)	84	(43.5)	11	(37.9)	18	(62.1)	1.4	(0.5–3.7)	0.474
2. Unsuccessful cutdown	97	(50.3)	96	(49.7)	4	(13.8)	25	(86.2)	9.7	(2.7–35.2)	0.001
3. Time spent using betel quid	129	(66.8)	64	(33.2)	13	(44.8)	16	(55.2)	2.7	(1.0–6.9)	0.042
4. Craving	124	(64.3)	69	(35.8)	10	(34.5)	19	(65.5)	2.8	(1.0–7.4)	0.043
5. Neglecting major roles	31	(16.1)	162	(83.9)	1	(3.5)	28	(96.6)	3.2	(0.4–27.1)	0.291
6. Social or interpersonal problems	121	(62.7)	72	(37.3)	5	(17.2)	24	(82.8)	8.8	(2.7–28.9)	<0.001
7. Abandoning or limiting activities	87	(45.1)	106	(54.9)	4	(13.8)	25	(86.2)	5.0	(1.4–17.7)	0.012
8. Hazardous use	92	(47.7)	101	(52.3)	2	(6.9)	27	(93.1)	13.8	(2.6–72.5)	0.002
9. Continued use despite knowing problems	127	(65.8)	66	(34.2)	6	(20.7)	23	(79.3)	6.6	(2.3–19.3)	0.001
10. Tolerance	111	(57.5)	82	(42.5)	10	(34.5)	19	(65.5)	2.0	(0.8–5.2)	0.146
11. Withdrawal	117	(60.6)	76	(39.4)	9	(31.0)	20	(69.0)	3.1	(1.2–8.2)	0.024
**Chewers with any domain symptom, yes/no**											
Impaired control	156	(80.8)	37	(19.2)	18	(62.1)	11	(37.9)	2.3	(0.8–6.1)	0.108
Social impairment	135	(70.0)	58	(30.1)	8	(27.6)	21	(72.4)	6.0	(2.1–16.8)	0.001
Risky use	135	(70.0)	58	(30.1)	6	(20.7)	23	(79.3)	7.8	(2.7–23.1)	<0.001
Pharmacological indicator	127	(65.8)	66	(34.2)	13	(44.8)	16	(55.2)	1.7	(0.7–4.4)	0.239
**Proportion of BUD in chewers, %**											
None (0–1 symptoms)	16.6				44.8				1.0		
Positive											
Mild (2–3 symptoms)	11.4				20.7				1.6	(0.4–6.1)	0.481
Moderate (4–5 symptoms)	14.0				17.2				1.9	(0.5–7.2)	0.347
Severe (≥6 symptoms)	58.0				17.2				7.7	(2.1–27.4)	0.002

*DSM*-*5*, Diagnostic and Statistical Manual of Mental Disorders, 5th Edition; OSCC, oral squamous cell carcinoma; BUD, betel quid use disorder. aOR, adjusted odds ratio. ^a^ aORs and *p* values were obtained from multivariable logistic regression models adjusted for sex, age, educational level, income, occupation, drink × years of drinking, and pack × years of smoking.

**Table 6:** Combined, conditional, and heterogeneous effect of *DSM*-*5* betel quid use disorder and pathological behavior on oral squamous cell carcinoma.

		OSCC (N = 233)		Control (N = 301)		Combined Effects		Conditional Effects		Heterogeneous Effects	
BUD Group	Pathological Behavior	No.	(%)	No.	(%)	aOR ^a^	(95% CI)	aOR ^a^	(95% CI)	aOR Ratio ^a,b^	*p*
Nonchewer		40	(17.2)	272	(90.4)	1.0	(ref.)				
**Chewer**											
**BUD status**	**Impaired control**										
None/Mild	None	36	(15.5)	11	(3.7)	8.0	(3.3–19.3)	1.0	(ref.)		
None/Mild	≥1 symptom	18	(7.7)	8	(2.7)	4.8	(1.7–13.8)	0.6	(0.2–2.0)	1.0	(ref.)
Moderate/Severe	None	1	(0.4)	0	(0.0)	NA		1.0	(ref.)		
Moderate/Severe	≥1 symptom	138	(59.2)	10	(3.3)	23.7	(10.3–54.7)	NA		NA	
**BUD status**	**Social impairment**										
None/Mild	None	38	(16.3)	18	(6.0)	4.3	(2.0–9.7)	1.0	(ref.)		
None/Mild	≥1 symptom	16	(6.9)	1	(0.3)	47.3	(5.4–411.9)	10.9	(1.2–101.4)	1.0	(ref.)
Moderate/Severe	None	20	(8.6)	3	(1.0)	7.0	(1.7–28.4)	1.0	(ref.)		
Moderate/Severe	≥1 symptom	119	(51.1)	7	(2.3)	32.0	(12.4–82.3)	4.6	(0.97–21.8)	0.4	0.530
**BUD status**	**Risky use**										
None/Mild	None	41	(17.6)	17	(5.7)	5.7	(2.6–12.7)	1.0	(ref.)		
None/Mild	≥1 symptom	13	(5.6)	2	(0.7)	12.5	(2.3–66.8)	2.2	(0.4–12.4)	1.0	(ref.)
Moderate/Severe	None	17	(7.3)	6	(2.0)	2.7	(0.9–8.6)	1.0	(ref.)		
Moderate/Severe	≥1 symptom	122	(52.4)	4	(1.3)	65.0	(20.1–210.0)	23.9	(5.2–109.8)	10.9	0.041 *
**BUD status**	**Pharmacological indicator**										
None/Mild	None	47	(20.2)	16	(5.3)	6.7	(3.1–14.7)	1.0	(ref.)		
None/Mild	≥1 symptom	7	(3.0)	3	(1.0)	5.7	(1.1–29.5)	0.9	(0.2–4.7)	1.0	(ref.)
Moderate/Severe	None	19	(8.2)	0	(0.0)	NA		1.0	(ref.)		
Moderate/Severe	≥1 symptom	120	(51.5)	10	(3.3)	19.3	(8.2–45.0)	NA		NA	

*DSM*-*5*, Diagnostic and Statistical Manual of Mental Disorders, 5th Edition; BUD, betel quid use disorder; OSCC, oral squamous cell carcinoma; aOR, adjusted odds ratio; ref., reference group; NA, non-appreciated due to no sample in the study group; *, *p* value < 0.05. ^a^ aORs and *p* values were obtained from multivariable logistic regression models adjusted for sex, age, educational level, income, occupation, drink × years of drinking, and pack × years of smoking. ^b^ aOR ratio was the ratio of the conditional aORs for moderate/severe BUD to none/mild BUD.

## Data Availability

The data are not publicly available due to ethical restrictions.

## Supplementary Materials

The following supporting information can be downloaded at: https://www.mdpi.com/article/10.3390/cancers14163974/s1.

## References

[1] Osborne P.G., Ko Y.C., Wu M.T., Lee C.H. (2017). Intoxication and substance use disorder to Areca catechu nut containing betel quid: A review of epidemiological evidence, pharmacological basis and social factors influencing quitting strategies. Drug Alcohol Depend..

[2] Warnakulasuriya S., Chen T.H.H. (2022). Areca nut and oral cancer: Evidence from studies conducted in humans. J. Dent. Res..

[3] Lee C.H., Ko A.M., Warnakulasuriya S., Yin B.L., Sunarjo, Zain R.B., Ibrahim S.O., Liu Z.W., Li W.H., Zhang S.S. (2011). Intercountry prevalences and practices of betel-quid use in south, southeast and eastern asia regions and associated oral preneoplastic disorders: An international collaborative study by asian betel-quid consortium of south and east Asia. Int. J. Cancer.

[4] Gupta P.C., Warnakulasuriya S. (2002). Global epidemiology of areca nut usage. Addict. Biol..

[5] (1985). Betel-quid and areca-nut chewing. IARC Monogr. Eval. Carcinog. Risk Chem. Hum..

[6] IARC Working Group on the Evaluation of Carcinogenic Risks to Humans (2004). Betel-quid and areca-nut chewing and some areca-nut derived nitrosamines. IARC Monogr. Eval. Carcinog. Risks Hum..

[7] Sharma D.C. (2003). Betel quid and areca nut are carcinogenic without tobacco. Lancet Oncol..

[8] Sari E.F., Prayogo G.P., Loo Y.T., Zhang P., McCullough M.J., Cirillo N. (2020). Distinct phenolic, alkaloid and antioxidant profile in betel quids from four regions of Indonesia. Sci. Rep..

[9] Tsai Y.S., Lee K.W., Huang J.L., Liu Y.S., Juo S.H., Kuo W.R., Chang J.G., Lin C.S., Jong Y.J. (2008). Arecoline, a major alkaloid of areca nut, inhibits p53, represses DNA repair, and triggers DNA damage response in human epithelial cells. Toxicology.

[10] Tsai Y.S., Lin C.S., Chiang S.L., Lee C.H., Lee K.W., Ko Y.C. (2011). Areca nut induces miR-23a and inhibits repair of DNA double-strand breaks by targeting FANCG. Toxicol. Sci..

[11] Gupta A.K., Tulsyan S., Thakur N., Sharma V., Sinha D.N., Mehrotra R. (2020). Chemistry, metabolism and pharmacology of carcinogenic alkaloids present in areca nut and factors affecting their concentration. Regul. Toxicol. Pharmacol..

[12] Lee C.H., Lee K.W., Fang F.M., Wu D.C., Tsai S.M., Chen P.H., Shieh T.Y., Chen C.H., Wu I.C., Huang H.L. (2012). The neoplastic impact of tobacco-free betel-quid on the histological type and the anatomical site of aerodigestive tract cancers. Int. J. Cancer.

[13] Hsu W.L., Chien Y.C., Chiang C.J., Yang H.I., Lou P.J., Wang C.P., Yu K.J., You S.L., Wang L.Y., Chen S.Y. (2014). Lifetime risk of distinct upper aerodigestive tract cancers and consumption of alcohol, betel and cigarette. Int. J. Cancer.

[14] Wu Y.H., Yen C.J., Hsiao J.R., Ou C.Y., Huang J.S., Wong T.Y., Tsai S.T., Huang C.C., Lee W.T., Chen K.C. (2016). A comprehensive analysis on the association between tobacco-free betel quid and risk of head and neck cancer in Taiwanese men. PLoS ONE.

[15] Lee Y.A., Li S., Chen Y., Li Q., Chen C.J., Hsu W.L., Lou P.J., Zhu C., Pan J., Shen H. (2019). Tobacco smoking, alcohol drinking, betel quid chewing, and the risk of head and neck cancer in an East Asian population. Head Neck.

[16] Ko A.M., Lee C.H., Ko A.M., Ko Y.C. (2020). Betel quid dependence mechanism and potential cessation therapy. Prog. Neuropsychopharmacol. Biol. Psychiatry.

[17] Lord G.A., Lim C.K., Warnakulasuriya S., Peters T.J. (2002). Chemical and analytical aspects of areca nut. Addict. Biol..

[18] Papke R.L., Horenstein N.A., Stokes C. (2015). Nicotinic activity of arecoline, the psychoactive element of “betel muts”, suggests a basis for habitual use and anti-inflammatory activity. PLoS ONE.

[19] Johnston G.A., Krogsgaard-Larsen P., Stephanson A. (1975). Betel nut constituents as inhibitors of gamma-aminobutyric acid uptake. Nature.

[20] Lodge D., Johnston G.A., Curtis D.R., Brand S.J. (1977). Effects of the areca nut constituents arecaidine and guvacine on the action of GABA in the cat central nervous system. Brain Res..

[21] Huang X., Liu Z., Mwansisya T.E., Pu W., Zhou L., Liu C., Chen X., Rohrbaugh R., Marienfeld C., Xue Z. (2017). Betel quid chewing alters functional connectivity in frontal and default networks: A resting-state fMRI study. J. Magn. Reson. Imaging.

[22] Huang X., Pu W., Liu H., Li X., Greenshaw A.J., Dursun S.M., Xue Z., Liu Z. (2017). Altered Brain Functional Connectivity in Betel Quid-Dependent Chewers. Front. Psychiatry.

[23] Lee C.H., Ko A.M., Warnakulasuriya S., Ling T.Y., Sunarjo, Rajapakse P.S., Zain R.B., Ibrahim S.O., Zhang S.S., Wu H.J. (2012). Population burden of betel quid abuse and its relation to oral premalignant disorders in South, Southeast, and East Asia: An Asian Betel-quid Consortium Study. Am. J. Public Health.

[24] Lee C.H., Ko A.M., Yen C.F., Chu K.S., Gao Y.J., Warnakulasuriya S., Sunarjo, Ibrahim S.O., Zain R.B., Patrick W.K. (2012). Betel-quid dependence and oral potentially malignant disorders in six Asian countries. Br. J. Psychiatry.

[25] Lee C.H., Chiang S.L., Ko A.M., Hua C.H., Tsai M.H., Warnakulasuriya S., Ibrahim S.O., Sunarjo, Zain R.B., Ling T.Y. (2014). Betel-quid dependence domains and syndrome associated with betel-quid ingredients among chewers: An Asian multi-country evidence. Addiction.

[26] Lee C.H., Ko A.M., Yang F.M., Hung C.C., Warnakulasuriya S., Ibrahim S.O., Zain R.B., Ko Y.C. (2018). Association of DSM-5 betel-quid use disorder with oral potentially malignant disorder in 6 betel-quid endemic Asian populations. JAMA Psychiatry.

[27] American Psychiatric Association (2013). Diagnostic and Statistical Manual of Mental Disorders.

[28] Hasin D.S., O’Brien C.P., Auriacombe M., Borges G., Bucholz K., Budney A., Compton W.M., Crowley T., Ling W., Petry N.M. (2013). DSM-5 criteria for substance use disorders: Recommendations and rationale. Am. J. Psychiatry.

[29] Lee C.H., Yang S.F., Peng C.Y., Li R.N., Chen Y.C., Chan T.F., Tsai E.M., Kuo F.C., Huang J.J., Tsai H.T. (2010). The precancerous effect of emitted cooking oil fumes on precursor lesions of cervical cancer. Int. J. Cancer.

[30] Mickey R.M., Greenland S. (1989). The impact of confounder selection criteria on effect estimation. Am. J. Epidemiol..

[31] Yang J., Wang Z.Y., Huang L., Yu T.L., Wan S.Q., Song J., Zhang B.L., Hu M. (2021). Do betel quid and areca nut chewing deteriorate prognosis of oral cancer? A systematic review, meta-analysis, and research agenda. Oral. Dis..

[32] Parkin D.M., Bray F., Ferlay J., Pisani P. (2005). Global Cancer Statistics, 2002. CA Cancer J. Clin..

[33] Torre L.A., Bray F., Siegel R.L., Ferlay J., Lortet-Tieulent J., Jemal A. (2015). Global Cancer Statistics, 2012. CA Cancer J. Clin..

[34] Ko Y.C., Huang Y.L., Lee C.H., Chen M.J., Lin L.M., Tsai C.C. (1995). Betel quid chewing, cigarette smoking and alcohol consumption related to oral cancer in Taiwan. J. Oral. Pathol. Med..

[35] Yen T.T., Lin W.D., Wang C.P., Wang C.C., Liu S.A. (2008). The association of smoking, alcoholic consumption, betel quid chewing and oral cavity cancer: A cohort study. Eur. Arch. Otorhinolaryngol..

[36] Lin W.J., Jiang R.S., Wu S.H., Chen F.J., Liu S.A. (2011). Smoking, alcohol, and betel quid and oral cancer: A prospective cohort study. J. Oncol..

[37] Benegal V., Rajkumar R.P., Muralidharan K. (2008). Does areca nut use lead to dependence?. Drug Alcohol Depend..

[38] Mirza S.S., Shafique K., Vart P., Arain M.I. (2011). Areca nut chewing and dependency syndrome: Is the dependence comparable to smoking? A cross sectional study. Subst. Abus. Treat. Prev. Policy.

[39] Lewis S., Pandey S., Salins N., Deodhar J., Patil V., Gupta T., Laskar S.G., Budrukkar A., Murthy V., Joshi A. (2021). Distress screening in head and meck cancer patients planned for cancer-directed radiotherapy. Laryngoscope.

[40] Kunz V., Wichmann G., Lehmann-Laue A., Mehnert-Theuerkauf A., Dietz A., Wiegand S. (2021). Screening for distress, related problems and perceived need for psycho-oncological support in head and neck squamous cell carcinoma (HNSCC) patients: A retrospective cohort study. BMC Cancer.

