# Supplementary material for: Effects of DSM-5 Betel-Quid-Related Symptoms, Pathological Behaviors, and Use Disorder on Oral Squamous Cell Carcinoma Risk
Source: Cancers (Basel). 2022 Aug 17;14(16):3974. doi: 10.3390/cancers14163974 (PMC9406538; doi:10.3390/cancers14163974)
Supplement: Supplementary file 1 [file cancers-14-03974-s001.zip › cancers-1847540-supplementary.pdf]

Supplementary Table S1. The types of betel-quid consumed in oral squamous cell carcinoma patients and controls among chewers

	OSCC (N = 193)		Control (N = 29)		Total (N = 222)	
**Types**	**No.**	**%**	**No.**	**%**	**No.**	**%**
AN only	12	6.2	3	10.3	15	6.8
AN + betel leaves	147	76.2	16	55.2	163	73.4
AN + flower of the *Piper betle*	7	3.6	2	6.9	9	4.1
Mixed use[a]	27	14.0	8	27.6	35	15.8

OSCC, oral squamous cell carcinoma; AN, areca nut.

[a]The mixed use of AN only, AN + betel leaves, and AN + flower of the *Piper betle*.

Supplementary Table S2. Adjusted odds ratios of oral squamous cell carcinoma associated with *DSM-5* betel-quid use disorder in chewers using different types of betel-quid

	AN-wrapped betel leaf				Others[a]			
	OSCC	Control			OSCC	Control		
***DSM-5* parameters**	**No.**	**No.**	**aOR[b]**	**(95% CI)**	**No.**	**No.**	**aOR[b]**	**(95% CI)**
**Betel-quid chewing**								
Non-chewer	40	272	1.0	Ref.	40	272	1.0	Ref.
**Betel-quid use disorder**								
Negative (0–1 symptoms)	22	6	10.4	(3.3–32.2)	10	7	2.0	(0.6–7.2)
Positive (≥2 symptoms)	125	10	21.1	(9.0–49.3)	36	6	12.7	(4.0–41.0)

DSM-5, Diagnostic and Statistical Manual of Mental Disorders, 5th Edition; OSCC, oral squamous cell carcinoma; aOR, adjusted odds ratio; Ref., reference group.

[a]Others included the users of AN only, AN + flower of the *Piper betle*, and mixed users.

[b]ORs were adjusted for sex, age, educational level, income, and occupation, alcohol drinking and cigarette smoking.
